# Reliability and Validity of Modified Algometer in Abdominal Examination

**DOI:** 10.1155/2016/3052954

**Published:** 2016-03-17

**Authors:** Seok-Jae Ko, Honggeol Kim, Seul-Ki Kim, Kyungmo Park, Jeungchan Lee, Beom-Joon Lee, Jayoung Oh, Kyungjin Lee, Jae-Woo Park

**Affiliations:** ^1^College of Korean Medicine, Kyung Hee University, Seoul 130-701, Republic of Korea; ^2^Department of Biomedical Engineering, Kyung Hee University, Yongin 449-701, Republic of Korea

## Abstract

*Objective*. Abdominal examination (AE) is one of the essential diagnostic methods in traditional Korean medicine that has been widely used for deciding treatment, cause, and prognosis of the disease. AE majorly depends on the experience of practitioners; therefore, standardization and quantification of AE are desperately needed. However, few studies have tried to objectify AE and established its standard. We assessed the reliability and validity of newly developed diagnostic device for AE called modified algometer (MA).* Methods*. Thirty-six subjects with functional dyspepsia were allocated into one of 2 groups according to gold standard of AE: epigastric discomfort without tenderness (*n* = 23) group or epigastric discomfort with tenderness (*n* = 13) group. Pressure pain threshold was evaluated at participants' epigastric region with algometer and MA. We assessed reliability and validity (sensitivity and specificity) and calculated optimal cutoff value.* Results*. MA showed high intertrial reliability (ICC 0.849; 0.703–0.923; *P* < 0.000) and validity (sensitivity: 76.92%; specificity: 60.87%), and cutoff value was 330.0 mmHg. Algometer and MA showed moderate correlation (*r* = 0.583, *P* ≤ 0.000).* Conclusion*. MA can be reliable and valid diagnostic device for AE and has the possibility of practical use for quantification and standardization of AE.

## 1. Introduction

Abdominal examination (AE) is one of the diagnostic methods in traditional Korean medicine (TKM) that enables TKM doctors to decide diagnosis and prognosis of the patients. AE was historically documented in the ancient medical book named “*Huang Di Neijing* (Yellow Emperor's Internal Classic)” and “*Shang Han Lun* (Treatise on Cold Diseases)” approximately 2,000 years ago [1]. The components of AE include abdominal distention, lumps, mass, pressure pain, growling, fluid sound, and skin temperature, and based on these items, 8 principle patterns (deficiency, excess, cold, heat, yin, yang, internal, and external) can be identified [2]. While western style of abdominal examination has features of mainly doctor-administered, objective, and sign-based diagnosis, TKM AE has features of mainly patient-administered, subjective, and symptom-based one [2].

“*Simhabi*” (SH) and “*Simhabikyung*” (SHK) are representative TKM diagnostic terminology of AE that are commonly detected in individuals with functional dyspepsia (FD). When TKM doctors applied a certain amount of pressure on patients' epigastric region, patients with SH complain of subjective discomfort without tenderness, while patients with SHK express discomfort with tenderness. According to SH or SHK, the treatment methods including herbal prescription can be divided obviously, and discrimination between them is very important in Korean medicine. However, the pressure intensity applied in AE has not been decided with a standardized diagnostic tool; therefore, the accuracy of AE depends mainly on practitioners' subjective experience which makes it difficult to judge identical diagnosis among TKM practitioners.

Algometer has long been used to measure pain of soft tissue associated with trigger points and shown to be an effective way of quantifying the pressure pain threshold (PPT) [3, 4]. There is evidence to support the reliability of algometer to measure the PPT of trigger points of neck, head, and shoulder [5], and another study provided further evidence of the reliability over muscle not having trigger points [6]. The application of algometer has been gradually extended to other areas, such as upper and lower abdomen [7, 8]. Recent study by Ko et al. [9] showed reliability and validity of algometer when it was applied in AE and successfully suggested the optimal cutoff value for discriminating between SH and SHK. The result of the study showed high interrater reliability (correlation coefficient range: 0.82–0.91) and intrarater reliability (intraclass correlation coefficient: 0.58–0.70) with 100% sensitivity, 54.54% specificity, and 1.8 kg/cm^2^ of optimal cutoff value between SH and SHK [9]. However, algometer still had the possible inconsistency of pressing skills which depend on proficiency of operators. Recently, Park et al. developed semiautomated algometer called modified algometer (MA) for maintaining a constant pressure/pressing speed and attaching/keeping a pressing site without operator's manipulation. If the MA is to be used to assess the effect of an intervention it must first be shown to be unequivocally reliable and valid, with the instrument making identical recordings on the same subject on repeated occasions and trustworthy results discriminating disease and normal.

Thus, the purpose of this study was to assess the within-session (intertrial) reliability of MA in the measurement of PPT over epigastric region in FD subjects and validity classifying SH and SHK with the optimal cutoff value between them. A robust analysis of MA measurement reliability and validity in AE has not been conducted previously.

## 2. Materials and Methods

### 2.1. Participants

One hundred and sixty-three participants from the age of 18 to 75, who complained of epigastric discomfort or pain, were screened for eligibility. They were recruited in Kyung Hee University Hospital at Gandong from July to November 2013. The participants had to fulfill the diagnostic criteria of FD based on Rome III [10] and meet other inclusion and exclusion criteria shown in the following part.

*Inclusion and Exclusion Criteria of the Study*

(i) Inclusion criteria are as follows:

1. the ages of 18 and 75 years,
2. individuals who meet functional dyspepsia (FD) definition of Rome III criteria,
3. individuals who check more than 40 mm on 100 mm visual analogue scale (VAS) for dyspeptic symptoms (VAS; 0: no symptom disturbance at all; 100: very severe),
4. normal esophagogastroduodenoscopy results within a year and diagnosed with FD by a specialist consultation,
5. individuals who receive no other treatments about dyspepsia during the study,
6. individuals who voluntarily agree with a study protocol and sign a written informed consent.

(ii) Exclusion criteria are as follows:

1. peptic ulcer or gastroesophageal reflux disease,
2. obvious signs of irritable bowel syndrome,
3. alarm symptoms (weight loss, black or tar stool, and dysphagia),
4. serious structural diseases (disease of heart, lung, liver, or kidney) or mental illness,
5. past surgical history related to the gastrointestinal tract except for appendectomy more than 6 months ago,
6. pregnancy or breastfeeding,
7. taking drugs which might affect gastrointestinal tract,
8. HIV-positive,
9. malabsorption or maldigestion,
10. other difficulties in attending the trial (e.g., paralysis, serious mental illness, dementia, drug addiction, time constraint, severe disorder in vision or hearing, illiteracy, etc.),
11. other diseases that could interfere with acupuncture treatment (e.g., clotting disorders or leukopenia, pacemaker, epilepsy, or anticoagulant therapy).

**Figure 1:**
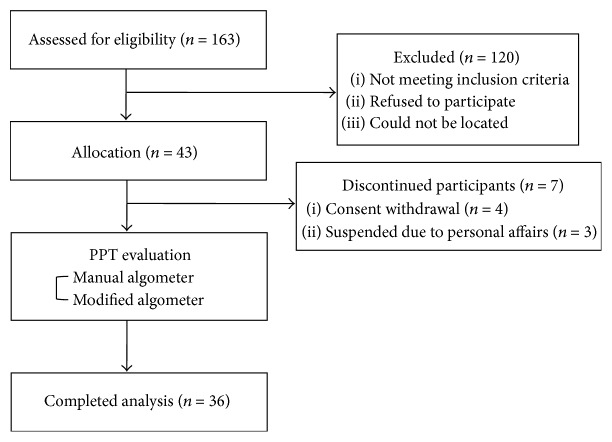
Flowchart of trial. MA: modified algometer.

Only the participants who understood the purpose of the study and signed an informed consent could participate in the study. They had rights to withdraw from the study voluntarily at any time. All adverse events during the study were recorded on the case report form in detail. Among total 43 participants who have passed the screening test, 7 participants dropped out, and 36 participants completed the study (Figure 1).

### 2.2. Study Protocol

Participants were diagnosed to one of 2 groups named SH group (*n* = 23) and SHK group (*n* = 13) by the consensus of 3 TKM practitioners with more than 3 years of clinical experience. A diagnosis of more than 2 TKM clinicians' consensus was regarded as a gold standard. Each practitioner was not aware of other 2 examiners' diagnosis during the session, and all participants were examined randomly and independently.

The examination was conducted as follows in accordance with the guidelines of the previous study [9]. (1) The participant was placed in the supine decubitus position on the bed. (2) All participants took a rest at least 5 minutes before the examination. (3) The TKM clinicians palpated the patients' abdomen with their right index, middle, and ring fingers [11]. As the pressure was gradually applied to patient's abdomen with practitioner's finger, patients expressed abdominal discomfort at a certain pressure level. Depending on the level of pressure, patients were diagnosed as either SH or SHK. (4) Temperature and humidity of the examination room were maintained 18°C and 40–50%, respectively.

This study was conducted in accordance with the protocol approved by International Review Boards in Kyung Hee University Hospital at Gangdong, Seoul (KHNMC-OH-IRB 2013-006), and with good clinical practice established by International Conference on Harmonization.

### 2.3. Evaluation of Overall Dyspepsia Symptom Using the Visual Analogue Scale (VAS)

Each participant was asked to score the severity of symptom on the 100 mm straight-line scale. The scale 0 on the left side meant absence of symptom, and the scale 100 on the right side meant the worst discomfort that he/she has ever had.

### 2.4. PPT Evaluation of Epigastric Region with Algometer and MA

PPT on the acupoint CV 14 [12] was measured by independent TKM practitioner with algometer and MA who was not aware of patient's TKM diagnosis. The mean value of 2 times measurement in algometer or MA, respectively, was regarded as an output value. Whole process of the measurement followed Fisher's guideline [13].

#### 2.4.1. Algometer

Algometer is a round-shaped device and the value rises as the amount of the pressure is applied. Examiner held the algometer perpendicular to the participant's skin and pressed it until the participant reports the first uncomfortable sensation which is defined as PPT [12, 14]. The algometer used in this study was Wagner FPK20® (Wagner Instruments, USA).

#### 2.4.2. Modified Algometer

**Figure 2:**
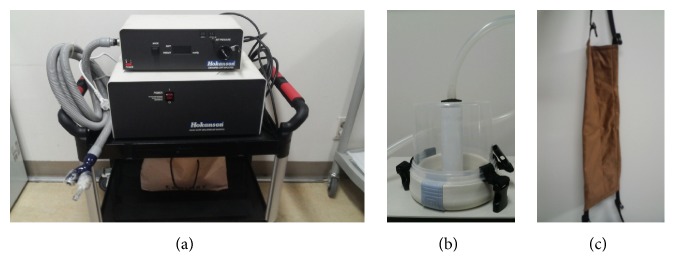
Modified algometer. (a) Pressure control device. (b) Circular cylinder to press. (c) Waistband to fix cylinder.

MA is a new device especially for evaluating PPT semiautomatically at epigastric region recently developed by Kyungmo Park (Figure 2). MA was applied along with the following order: the power switch was turned on and scale was adjusted to zero. Cylindrical piston in the machine was attached to the epigastric area of the participants (CV 14) and fixed with belt in order not to move around during process of pressing. The examiner slowly turned the lever around at a constant speed, thereby increasing the pressure. The pressure was transmitted to the pneumatic piston pressing the subject. When the subject complained of discomfort at pressure site (CV 14), the examiner stopped to turn the lever and read the scale called PPT. Then the examiner pressed cuff button and the pressure fell immediately. The examiner reevaluated PPT again in the same way after one minute's rest.

### 2.5. Evaluation of Intertrial Reliability and Validity on MA

#### 2.5.1. Intertrial Reliability

As MA is a semiautomated device where the value does not depend on operator, therefore we evaluated only trial to trial reliability. The trials were repeated with one-minute interval and the correlation of PPT value between the first and second trials was analyzed.

#### 2.5.2. Validity

Validity means the degree of match between assessed value by device and the presence (or absence) of actual disease. It is estimated by sensitivity and specificity representing the accuracy of the test [15].

A trade-off between two measures can be represented graphically as a receiver operating characteristic (ROC) curve [16]. In order to obtain the sensitivity and specificity of the test, the optimal cutoff value between SH and SHK was acquired through ROC curve analysis. In ROC curve, consensus of clinicians was considered as a gold standard, and the PPT value measured by algometer or MA was considered as a test variable. We calculated the sensitivity and specificity from 2 × 2 table and also could obtain the positive predictive value and the negative predict value.

### 2.6. Statistical Analysis

All data of continuous variables were presented as mean ± standard deviation (SD), and all data of categorical variables were presented as percentages (*n*, %). The mean values of continuous variable or the data of categorical variable between 2 groups was compared by two sample *t*-test or Fisher's exact test, respectively. Correlation between continuous variables in each group was calculated by Pearson's correlation analysis. The *P* value < 0.05 was considered to be statistically significant and statistical analysis was done by PASW Statistics 18.0 (SPSS Inc., USA).

#### 2.6.1. Statistical Analysis for Intertrial Reliability

Agreement between measurements was analyzed by the intraclass correlation coefficient (ICC 2,1) with 95% confidence intervals [17]. The standard error of the mean (SEM) was calculated from the square root of the mean square of error derived from the analysis of variance [18]. The smallest real difference (SRD) with 95% confidence was calculated as 1.96 × √2 × SEM. The SEM% and SRD% were calculated to represent measurement error in relative terms [19]. All analyses were performed in the PASW Statistics 18.0 (SPSS Inc., USA).

#### 2.6.2. Statistical Analysis for Validity

Subjects were categorized according to whether they were SH or SHK based on TKM diagnosis and paired  *t*-tests were used to compare PPTs between SH and SHK. Number of subjects classified by TKM diagnosis (SH and SHK) and optimal cutoff value were cross-tabulated, and sensitivity, specificity, and positive predictive values and negative predictive values were determined for MA. Analyzing the ROC curve and determining the optimal cutoff value were carried out using MedCalc 12.3.0 (MedCalc software bvba, Belgium).

## 3. Results

### 3.1. Baseline Characteristics of the Subjects

Finally, 36 participants completed the trials. The baseline characteristics of the participants such as age and height were well-balanced between 2 groups shown in Table 1.

### 3.2. Comparison of VAS and PPT between Groups

The differences in VAS score between 2 groups were not statistically significant (Table 2). The differences of PPT were statistically significant, evaluated by both algometer (*P* = 0.003) and MA (*P* = 0.019, Table 2).

### 3.3. Intertrial Reliability and Validity Evaluation of MA

#### 3.3.1. Intertrial Reliability

In trial to trial reliability assessment, we found high ICC (0.849; 0.703–0.923; *P* < 0.000). The first trial of evaluating PPT with MA was 346.83 ± 61.87, SEM% value in first trial was 3%, and SRD% was 8%. The second trial of evaluating PPT with MA was 341.75 ± 86.64, and SEM% value was 4%, and SRD% was 12%.

#### 3.3.2. Validity

**Figure 3:**
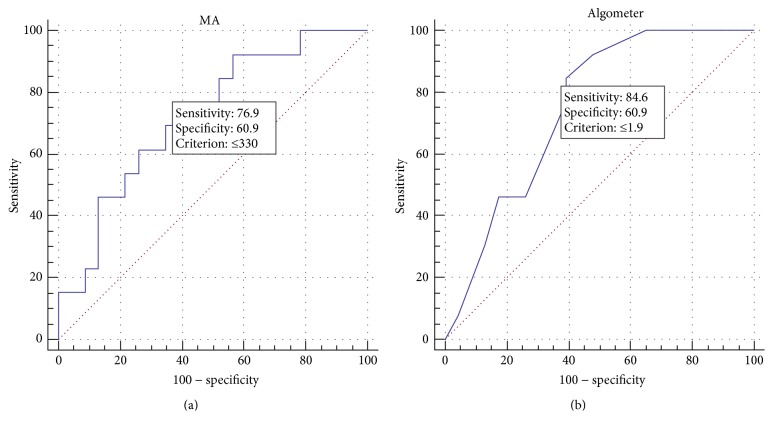
ROC curve of PPT value at CV 14 by MA (a) and algometer (b). ROC: receiver operating characteristic; PPT: pressure pain threshold; MA: modified algometer.

The optimal cutoff value of MA between SH and SHK based on ROC curve analysis was determined as 330.0 mmHg, and the sensitivity and specificity were 76.92% and 60.87%, respectively (Figure 3, Table 3). The optimal cutoff value of algometer was 1.9 kg/cm^2^, and the sensitivity and specificity were 84.62% and 60.87%, respectively (Figure 3).

### 3.4. Analysis of the Correlation between Algometer and MA

Pearson's correlation coefficient of PPT value between algometer and MA was *r* = 0.583, and explanatory power was *r* ^2^ = 0.340. These results showed a moderate positive correlation between algometer and MA with statistical significance (*P* ≤ 0.000).

## 4. Discussions

In the current study, we attempted to standardize and quantify AE diagnosis with newly developed MA which is an advanced device compared with conventional algometer in terms of consistency and validity. As a result of this study, MA has successfully distinguished SH and SHK with a statistical significance and suggested the optimal cutoff value between them. MA showed 76.92% of sensitivity and 60.87% of specificity and was regarded as a trustworthy diagnostic device with high intertrial reliability. MA also correlated with manual algometer in PPT.

Ko et al. have widened the application of a manual algometer that was used originally for measuring PPT in subcutaneous muscles [9]. In the previous study, algometer successfully differentiated SH and SHK with high reliability and validity, which was the first step for the development of quantitative indicator of AE [9]. However, algometer has some limitations in measuring PPT in abdomen, because the device should be operated by experienced practitioners and the method of putting pressure on abdomen was slightly different depending on them. Additionally, the speed of adding pressure might not be consistent when pressing the patients' certain abdominal point using manual algometer, and for that reason, the response timing of patient's complaint about abdominal discomfort might not match that of the real PPT. Generally, PPT values increase with delayed response of evaluator to patient's reaction, and the higher velocity of pushing pressure results in higher PPT values [8]. Therefore, MA was considered as a more advanced diagnostic device for AE that had several strong points compared with a conventional manual algometer. (1) The location of pushing pressure was fixed with waistband that coiled the waist in order that the cylinder should not move around during evaluation (Figure 2). The band was tight enough to fix it even on obese abdomen and smooth surface. (2) Instead of pushing pressure manually, the operator just rotated the valve to increase pressure that the constant speed could be possible and set pressure could be put immediately with one button, which enabled more accurate measurement of PPT. Though the operator was not a skilled person, the pressure or PPT could be easily measured, and measured PPT became more reliable. (3) The operator could catch the exact response timing of patient's abdominal discomfort more easily due to semiautomated procedure. Although the operator was difficult to precisely evaluate the real initial PPT for manual algometer, MA could make it easier, so the operator simply stopped to rotate pressure valve of MA and read the screen of pressure level when the patient indicated initiation of pain. Therefore, MA can be regarded as an advanced device for AE, because the process of applying pressure is semiautomated, so the limitations of manual algometer can be minimized.

In the analysis of mean PPT values at CV 14, the mean PPT value of SH was significantly higher than that of SHK using both devices. This result is the same as that of the previous study [9] and it means that the patients with SHK feel pain easily with lower pressure on epigastrium in both studies.

In intertrial reliability assessment, ICC between the first and second trials was high (0.849; 0.703–0.923; *P* ≤ 0.000). The previous study using original algometer showed 0.82 or 0.87 coefficient of correlation in test-retest reliability and 0.58 of ICC in interrater correlation of PPT. Compared with the previous study, MA and algometer were little different in reliability, and MA could be more accurate in interrater reliability due to semiautomated process of evaluation. The SEM% of original algometer in the present study was 33%, and SEM% of MA showed 3%. This value showed that MA had lower level of error range compared with original algometer.

The sensitivity of MA was lower than that of algometer, and specificity was the same (current study) or higher than that of previous study. The optimal cutoff value of manual algometer by previous study (1.9 kg/cm^2^) was very similar in current study (1.8 kg/cm^2^). Therefore, 330.0 mmHg measured by MA as the optimal cutoff pressure level can be considered the same level in case of above values measured by manual algometer (330.0 mmHg = 1.92 kg/cm^2^). Considering that examiners with more than 3 years of clinical experiences measured PPT in the previous study and comparatively unskilled examiner evaluated PPT in this study, it can be suggested that the validity of MA in terms of diagnosis was clearly confirmed. MA was also moderately correlated with manual algometer according to Pearson's correlation coefficient of PPT analysis.

Based on the results mentioned above, we could conclude that the PPT measurement with MA for the diagnosis in AE has high intertrial reliability and validity as a new quantified method enough to replace the conventional AE or previous study on the manual algometer by the TKM clinician.

In spite of many strong points of MA, MA still has several limitations. (1) MA is big and heavy; therefore, it is difficult to move around without carrying implement. (2) MA in this study uses pneumatic method to put the pressure which makes it less precise to calculate PPT than cyclostyle device. However, MA in this study was made up of nonmetallic materials, so that it has an advantage of wide application to various circumstances including fMRI study. (3) MA is still a semiautomated device; therefore, it is needed to develop fully automated device considering pressing method and reading patient's response. Moreover, future study should assess the reliability of test-retest at long interval such as 1 week, interrater reliability of MA, and reliability or validity of PPT targeting patients with mild dyspeptic symptoms (VAS < 40 mm). The large sample size and equal number of both groups should be also considered.

## 5. Conclusion

MA showed high intertrial reliability and validity for AE. SH and SHK that are important diagnosis in TKM can be differentiated with 330 mmHg of the optimal cutoff value with MA. MA presented moderate correlation with conventional algometer and advancement in terms of consistency and objectivity. MA will be applied variously in TKM diagnosis and provide basis for quantification and standardization of AE.

## Figures and Tables

**Table 1:** Baseline characteristics of subjects.

Total (*n *= 36)	Mean ± SD	Range	
Age (y)	50.61 ± 12.00	24–69	
Height (cm)	161.78 ± 7.21	149.8–178.4	
Weight (kg)	54.99 ± 8.58	42.2–75.3	
BMI (kg/m^2^)	20.98 ± 2.79	16.2–28.8	

Baseline characteristics of each group			
	SH (*n *= 23)	SHK (*n *= 13)	*P* value

Age (y)	51.57 ± 11.07	48.92 ± 13.82	0.534
Height (cm)	162.05 ± 7.87	161.31 ± 6.15	0.771
Weight (kg)	55.08 ± 9.92	54.83 ± 5.83	0.935
BMI (kg/m^2^)	20.90 ± 2.99	21.14 ± 2.51	0.806

Sex (M, %)	30.43	7.74	0.122
P/H (%)	78.26	69.23	0.414
Surgery (%)	39.13	46.20	0.474
PD (%)	13.04	23.13	0.369
NPD (%)	0	0	—
Drinking (%)	47.83	23.13	0.134
Smoking (%)	8.70	0.0	0.402
Coffee (%)	56.52	61.53	0.526

Age, height, weight, and BMI are analyzed by two-sample *t*-test. Sex, P/H, surgery, PD, NPD, drinking, smoking, and coffee are analyzed by Fisher's exact test. The data of age, height, weight, and BMI in each group are presented by mean ± SD. SD: standard deviation; BMI: body mass index; P/H: physical history; PD: prescription drug; NPD: nonprescription drug; SH:* Simhabi*; SHK: *Simhabikyung*.

**Table 2:** Comparison of VAS for overall dyspepsia and PPT values at CV 14 by algometer and MA between 2 groups.

	SH (*n *= 23)	SHK (*n *= 13)	*P* value
VAS	44.91 ± 15.94	46.31 ± 11.51	0.784
PPT			
Algometer (kg/cm^2^)	2.12 ± 0.49	1.72 ± 0.23	0.003^*∗∗*^
MA (mmHg)	356.15 ± 68.11	302.77 ± 58.14	0.019^*∗*^

Analyzed by two-sample *t*-test. All data are presented by mean ± SD.* P* value < 0.05 is considered as statistically significant. ^*∗∗*^ *P* < 0.01; ^*∗*^ *P* < 0.05. SH:* Simhabi*; SHK: *Simhabikyung*; VAS: visual analog scale; PPT: pressure pain threshold; MA: modified algometer.

**Table 3:** Two-by-two table using a new test at CV 14 for abdominal examination, and actual SH or SHK as the criterion test.

	Abdominal examination	
	SHK	SH
PPT value by MA		
≤330.0	10	9
>330.0	3	14
Sensitivity	76.92%	
Specificity	60.87%	
Positive predictive value	52.63%	
Negative predictive value	82.35%	

Sensitivity: 0.77 (10/13); specificity: 0.61 (14/23); positive predictive value: 0.53 (10/19); negative predictive value: 0.82 (14/17). SH: *Simhabi*; SHK: *Simhabikyung*; PPT: pressure pain threshold; MA: modified algometer.
